# Viable supply chain model: integrating agility, resilience and sustainability perspectives—lessons from and thinking beyond the COVID-19 pandemic

**DOI:** 10.1007/s10479-020-03640-6

**Published:** 2020-05-22

**Authors:** Dmitry Ivanov

**Affiliations:** Department of Business Administration, Berlin School of Economics and Law, 10825 Berlin, Germany

**Keywords:** Supply chain, Viability, Resilience, Sustainability, Lean, Agility, COVID-19, Pandemic, Recovery, Viable supply chain

## Abstract

Viability is the ability of a supply chain (SC) to maintain itself and survive in a changing environment through a redesign of structures and replanning of performance with long-term impacts. In this paper, we theorize a new notion—the viable supply chain (VSC). In our approach, viability is considered as an underlying SC property spanning three perspectives, i.e., agility, resilience, and sustainability. The principal ideas of the VSC model are adaptable structural SC designs for supply–demand allocations and, most importantly, establishment and control of adaptive mechanisms for transitions between the structural designs. Further, we demonstrate how the VSC components can be categorized across organizational, informational, process-functional, technological, and financial structures. Moreover, our study offers a VSC framework within an SC ecosystem. We discuss the relations between resilience and viability. Through the lens and guidance of dynamic systems theory, we illustrate the VSC model at the technical level. The VSC model can be of value for decision-makers to design SCs that can react adaptively to both positive changes (i.e., the agility angle) and be able to absorb negative disturbances, recover and survive during short-term disruptions and long-term, global shocks with societal and economical transformations (i.e., the resilience and sustainability angles). The VSC model can help firms in guiding their decisions on recovery and re-building of their SCs after global, long-term crises such as the COVID-19 pandemic. We emphasize that resilience is the central perspective in the VSC guaranteeing viability of the SCs of the future. Emerging directions in VSC research are discussed.

## Introduction

**Fig. 1:**
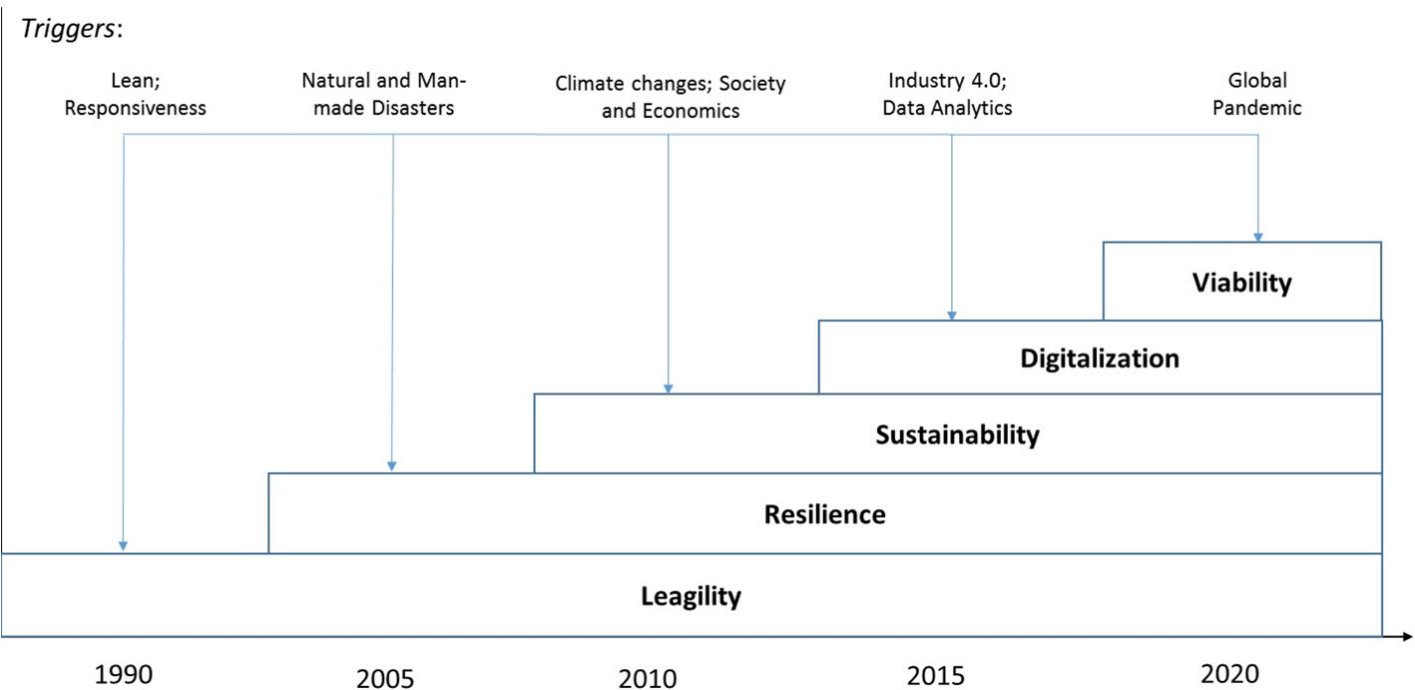
Transformation of major SC management research angles over time

Supply chains (SC) are a backbone of economies and society, and largely interact with nature. The interactions in these SC ecosystems are very complex and triggered by mutual interrelations and feedbacks between SCs, nature, society, and the economy. Being initially developed in the veins of leanness and agility, and their combination as leagility (Christopher and Towill 2000; Lee 2004; Goldsby et al. 2006; Eckstein et al. 2015; Gunasekaran et al. 2016; Dubey et al. 2018; Fadaki et al. 2020), SC research has been extended by the perspectives of resilience (Christopher and Peck 2004; Blackhurst et al. 2005; Tang 2006; Sawik 2011; Spiegler et al. 2012; Dubey et al. 2019a; Hosseini et al. 2019a; Wood et al. 2019) and sustainability (Seuring 2013; Brandenburg and Rebs 2015; Dubey et al. 2015; Allaoui et al. 2019) followed by the advanced utilization of digital technologies and Industry 4.0 (Wamba et al. 2015; Ivanov et al. 2016; Choi et al. 2018; Dolgui et al. 2020, 2020a; Dubey et al. 2019b; Ivanov et al. 2019b; Ghadge et al. 2020; Queiroz et al. 2020) (Fig. 1).

The current state-of-the-art state-of-the-art results in SC management stem from a number of remarkable transformations. In Fig. 1, these transformations are framed in a historical perspective. Being lean, responsive, and globalized in structural designs, SCs have also learned a great deal about how to act in line with nature and societal interests (i.e., become sustainable), how to strengthen their resilience during disruptions triggered by severe natural or man-made disasters, how to recover and manage the ripple effects (Ivanov et al. 2014a, b; Dolgui et al. 2018; 2020b), and how to utilize the advantages of digital technologies in SC management.

However, in 2020, the leagility, resilience, and sustainability of SCs have been put to the test. SCs worldwide have experienced an unprecedented series of shocks caused by the COVID-19 virus outbreak and global pandemic, a new instigator of SC disruptions quite unlike any seen in recent times (Chesbrough 2020; Choi 2020; Currie et al. 2020; Ivanov 2020a; Ivanov and Dolgui 2020b; Ivanov and Das 2020; Sarkis et al. 2020). The COVID-19 outbreak and global pandemic have immensely affected all areas of the economy and society raising a series of completely novel decision-making settings for SC researchers and practitioners:

- Have the established SC resilience measures (e.g., anticipatory and coping mechanisms such as risk mitigation inventories, subcontracting capacities, backup supply and transportation infrastructures, omni-channel and data-driven, real-time monitoring and visibility systems) (Craighead et al. 2007; Ho et al. 2015; Hosseini et al. 2019) helped the companies to survive and recover through the pandemic?
- Could the SCs quickly adapt and serve to secure the minimal survival needs of society and economies (WEF 2020)?
- How can digital technologies help mitigate the effects of severe risks during globally propagating disruptions such as epidemic outbreaks (e.g., COVID-19) disrupting global SCs (Araz et al. 2020; Ivanov 2020a)?

For some SCs, demand has drastically increased and supply was not able to cope with that situation (e.g., face masks, hand sanitizer, disinfecting spray). As such, the question of market and society survivability was raised. For other SCs, demand and supply have drastically dropped resulting in production stops (e.g., automotive industry), the danger of bankruptcies, and the necessity of governmental supports. Here the questions of SC survivability again arose. It is evident that both of these questions go beyond the existing state of the art in SC leagility, sustainability, and resilience because they cannot be resolved individually within each of these perspectives and require integrated frameworks and an extension when long-term, severe global disruptions affect all elements of SC ecosystems (i.e., businesses, society, nature, and economies).

Despite the considerable progress in the state of the art and practical applications in each of the individual frameworks (i.e., agile, lean, sustainable, resilient, and digital SC) (Altay et al. 2018; Bier et al. 2020; Blackhurst et al. 2011; Brandenburg and Rebs 2015; Choi et al. 2018; Das et al. 2006; Dolgui et al. 2018; Dubey et al. 2015; Govindan et al. 2016; DuHadway et al. 2019; Hosseini et al. 2019a; Ivanov 2018b; Ivanov and Dolgui 2019; Ivanov et al. 2019a; Tang and Veelenturf 2019; Wamba et al. 2017), there appears to be a lack of a holistic approach around these individual frameworks which could conceptually guide their roles and interplays as an integrated whole. In addition, a large body of humanitarian logistics and SC literature can be considered to close the existing research gap in the literature on commercial SC disaster-tolerance (Dubey et al. 2019c, d, Fosso Wamba 2020). Besides, the issues of SC survivability have not been studied intensively but were recognized as crucial topics following the COVID-19 pandemic propagations (Choi et al. 2020; Haren and Simichi-Levi 2020; Ivanov 2020a, b; Ivanov and Dolgiu 2020; Ivanov and Das 2020; Ni et al. 2020).

The example of the COVID-19 pandemic shows that in cases of extraordinary events, SC resistance to disruption needs to be considered at the scale of survivability or viability to avoid SC and market collapses and secure the provision of goods and services. According to Ivanov and Dolgui (2020b), “viability is a behavior-driven property of a system with structural dynamics. It considers system evolution through disruption-reaction balancing in the open system context. The viability analysis is survival-oriented at a long-term scale.” Ivanov (2018b, p. 59) defines SC viability as an “ability to survive and exist after a disruption [….] with the re-design of the supply chain structure and re-planning economic performance with long-term impacts.” This SC ability to meet the demands of surviving in a changing environment follows the notions of the Viable System Model by Beer (1985) developed for intercompany perspective and ecology modeling angles (Aubin 1991). Viability in this context can be understood considering approaches in ecological modeling. Ecological modeling is a research area concerned with the analysis of ecosystems in dynamics (Gross et al. 2004, 2009). Recent literature points to a resemblance of SCs to ecosystems (Byrne et al. 2018; Gross et al. 2018; Demirel et al. 2019; Nair and Reed-Tsochas 2019).

To close the research gaps described above, in this study we theorize a new notion—the viable supply chain (VSC). Our contribution lies in conceptualization of a VSC model spanning three perspectives, i.e., agility, resilience, and sustainability. The principal ideas of the VSC model are adaptable structural SC designs for supply–demand allocations and, most importantly, establishment and control of adaptive mechanisms for transitions between the structural designs. Further, we demonstrate how the VSC components can be categorized across organizational, informational, process-functional, technological, and financial structures. Moreover, our study offers a VSC framework within an SC ecosystem. Through the lens and guidance of dynamic systems theory, we illustrate the VSC model at the technical level. The VSC model can be of value for decision-makers to design SCs that can react adaptively to both positive changes (i.e., the agility angle) and be able to absorb negative disturbances, recover and survive during short-term disruptions and long-term, global shocks with societal and economical transformations (i.e., the resilience and sustainability angles). The VSC model can help firms in guiding their decisions on recovery and re-building of their SCs after global, long-term crises such as the COVID-19 pandemic. We emphasize that resilience is the central perspective in the VSC guaranteeing viability of the SCs of the future. Emerging directions in VSC research are discussed.

The rest of this study is organized as follows. In Sect. 2, we elaborate on the viable SC ecosystem framework, discuss the relations between SC resilience and viability, describe the VSC model, and present the multi-structural view of the VSC model. In Sect. 3, we illustrate the SC viability formation through the lens of dynamic systems theory. Section 4 is created to map out some directions of a future research agenda in VSCs. We conclude the paper in Sect. 5 by summarizing the most important insights.

## Viable supply chain model

### Definition

The concept of viability has been extensively developed in ecology, biological systems (Aubin 1991) and cybernetics (Beer 1985). Viability is the highest analysis level for SC reactions to disturbances which is based upon stability, robustness and resilience as follows:

- *Stability* The ability to return to a pre-disturbance state and ensure a continuity (Ivanov and Sokolov 2013; Demirel et al. 2019)
- *Robustness* The ability to withstand a disruption (or a series of disruptions) to maintain the planned performance (Nair and Vidal 2011; Simchi-Levi et al. 2018)
- *Resilience* The ability to withstand a disruption (or a series of disruptions) and recover the performance (Spiegler et al. 2012; Hosseini et al. 2019a; Zhao et al. 2019).
- *Viability* The ability to maintain itself and survive in a changing environment over a long period of time through a redesign of the structures and replanning of economic performance with long-term impacts (Ivanov 2018b, p. 59; Ivanov and Dolgui 2020b).

Generally speaking, SC reactions to disturbances have been mostly studied at the semantic network analysis level. Network topologies, structural properties, complexity factors, and node/arc criticality dominate this research stream (Basole and Belami 2014; Kim et al. 2015; Brintrup et al. 2015; Sawik 2017; Macdonald et al. 2018; Yoon et al. 2018; Scheibe and Blackhurst 2018; Pavlov et al. 2018; Ojha et al. 2018; Ivanov 2018a; Ivanov et al. 2019a; Dolgui et al. 2018; Ivanov and Dolgui 2019; Li et al. 2019; Pavlov et al. 2019a, b).

The major principles of viability modeling across the disciplines are survival orientation, the absence of explicit time windows in analysis, and ecosystem focus. As such, we define a VSC as follows:

Viable supply chain (VSC) is a dynamically adaptable and structurally changeable value-adding network able to (i) react agilely to positive changes, (ii) be resilient to absorb negative events and recover after the disruptions, and (iii) survive at the times of long-term, global disruptions by adjusting capacities utilizations and their allocations to demands in response to internal and external changes in line with the sustainable developments to secure the provision of society and markets with goods and services in long-term perspective.

This understanding of VSC spans various management and organizational principles from the systems, information, organization, and network theories and can be considered through the lens of these theories. Beer’s Viable System Model (Beer 1985) allows us to understand how interconnected operations communicate with changing market environments and meta-systems such as markets, policy, and society. Through the lens of viability, the Beer’s model builds upon an analogy with the human organism as the most advanced, survival-oriented complex system. According to Ashby’s law of requisite variety (Ashby 1956), the situational variety should be balanced by the response variety of the controller or “only variety absorbs variety.” This law can be considered as one of the VSC pillars in the development of highly diversified and decentralized systems able to respond to increasing variety in the external systems such as new market models (e.g., omnichannel), new business models (e.g., circular economy), positive disruptions (e.g., innovations), and negative disruptions (e.g., natural catastrophes), to build resilient and sustainable operational systems. Moreover, VSC poses open system context analysis. An open system (Mesarovic and Takahara 1975; Casti et al. 1979) is a system that has interactions with the environment and evolves based on these interactions. The major characteristics of open systems are control, self-adaptation, and self-organization (von Bertallanfy 1969), which can be seen as future-leading management principles for VSC.

### Viable supply chain model

**Fig. 2:**
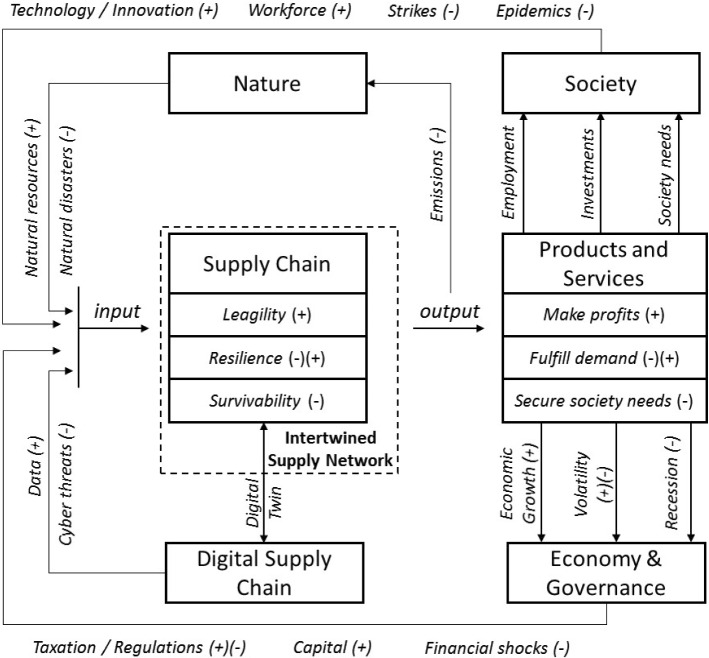
Viable supply chain ecosystem framework

**Fig. 3:**
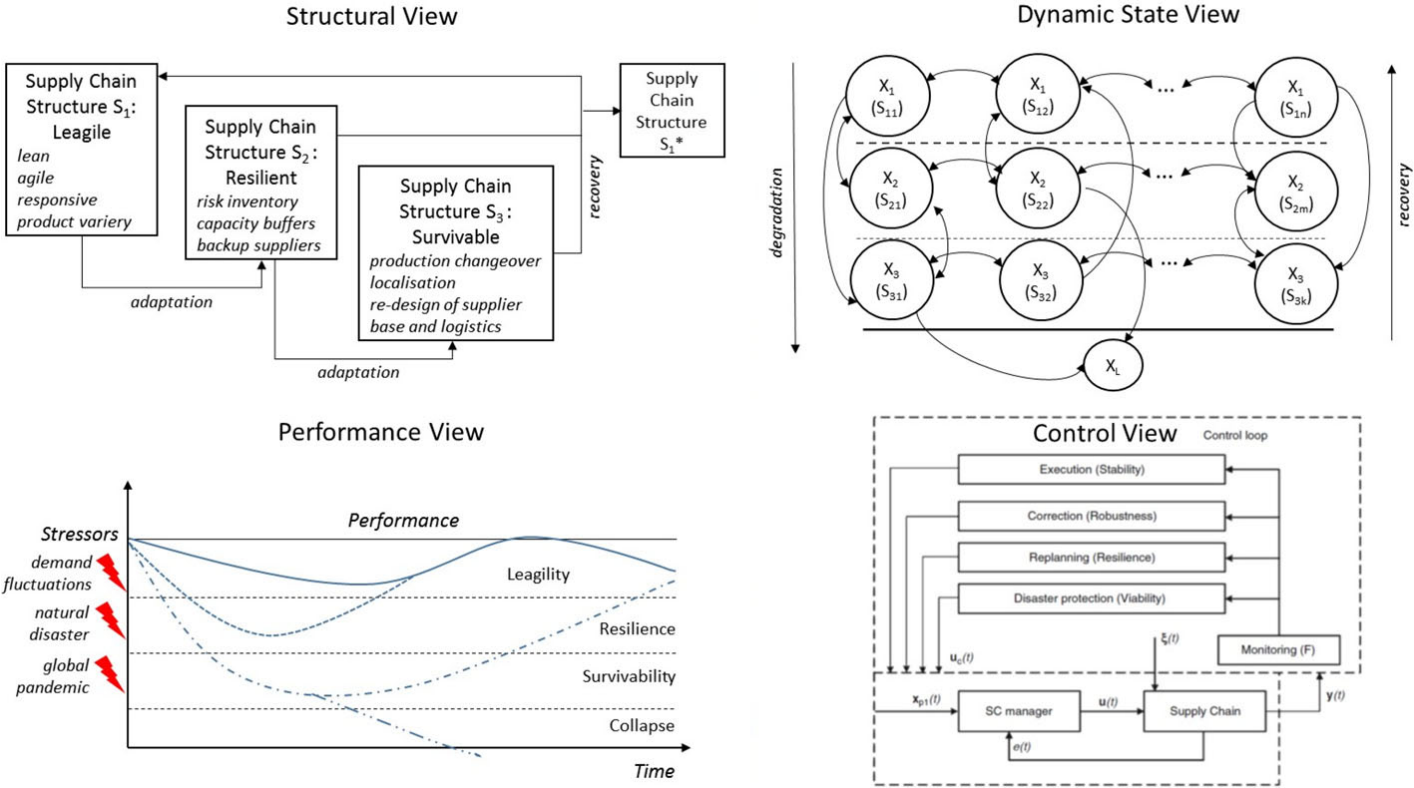
Viable supply chain model

**Fig. 4:**
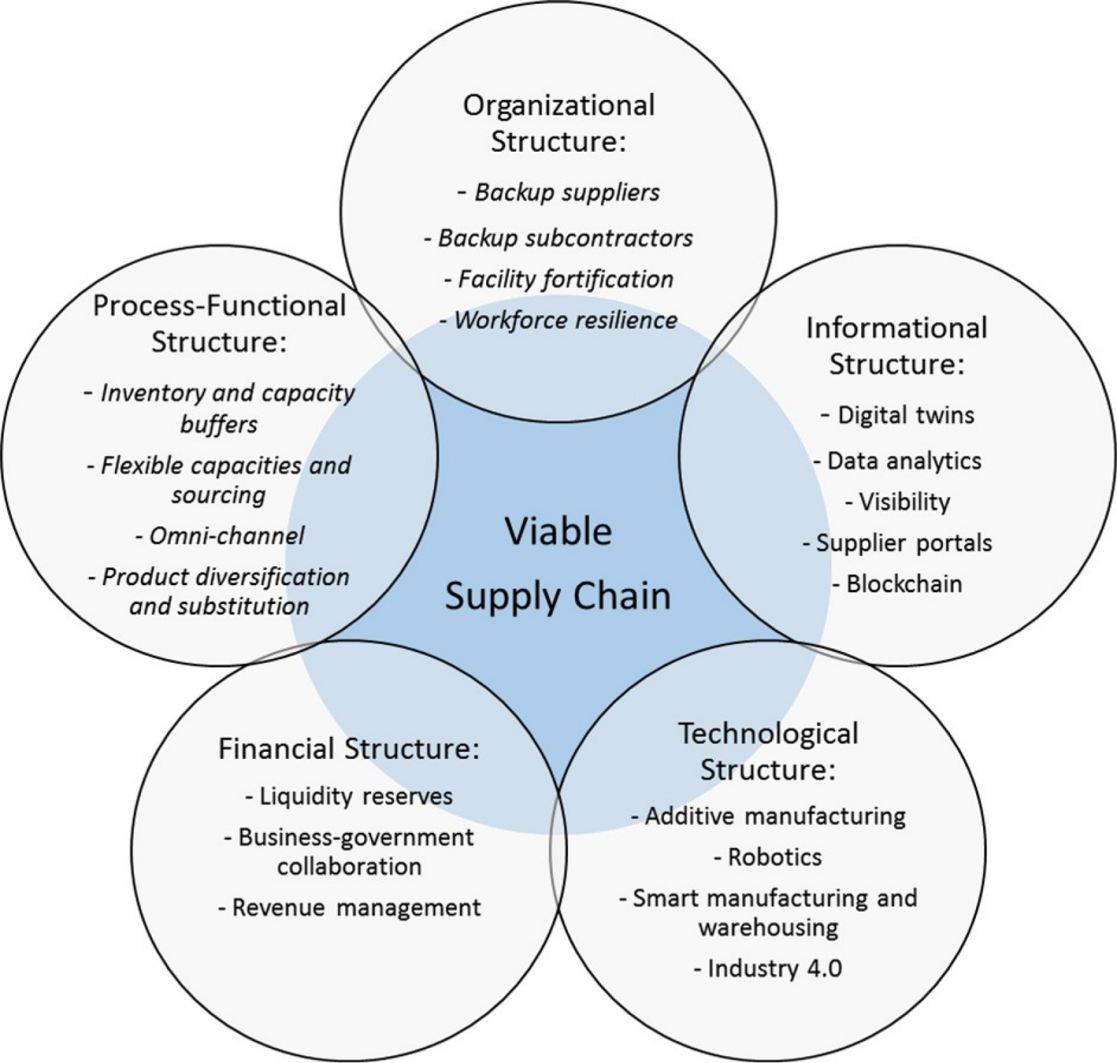
Multi-structural VSC view

In this section, we present the VSC model. We begin with a framework of an SC ecosystem that spans three feedback cycles of leagility, resilience, and survivability (Fig. 2). Subsequently, the VSC model is presented (Fig. 3). We demonstrate how the VSC components can be categorized across organizational, informational, process-functional, technological, and financial structures (Fig. 4). Finally, we discuss on the relations between resilience and viability at the generalized level.

#### Viable supply chain ecosystem framework

The VSC ecosystem framework is comprised of the following components (Fig. 2):

- the SC itself.
- the intertwined supply network (ISN), which is an “entirety of interconnected supply chains which, in their integrity secure the provision of society and markets with goods and services” (Ivanov and Dolgui 2020b).
- society
- nature
- economy and governance
- digital SC, which—in a combination with the physical SC—represents a cyber-physical system framing a digital SC twin (Panetto et al. 2019; Ivanov and Dolgui 2020a).

The VSC ecosystem framework in Fig. 2 is built around three feedback cycles:

- A positive feedback cycle (+), which refers to disruption-free SC operations with the main objective to maximize profitability.
- A volatile feedback cycle (+)(−), which refers to disruptions and recovery within the SC resilience scope with the main objective to restore system operations and performance, and
- A survivability feedback cycle (−), which refers to the long-term, global crises with the main objective to maintain the SC existence and to secure the provision of society with the SC’s products or services.

An SC can be considered viable if it is able to maintain an ecosystem balance (i.e., achieve homeostasis) within all three feedback cycles. The positive cycle is concerned with profitability, developments, investments, efficiency, agility, and responsiveness. This is the time to use the advantages of technological developments, innovations, and market growth to react to positive changes. The volatile cycle is concerned with sustaining and recovering from the disruptions to fulfill the demand. The cycle of survivability is intended to secure the provision of economy and society with the products and services.

Within each of the three feedback cycles in the SC ecosystem, there are internal positive and negative feedbacks. For example, the interactions of the SC and nature are concerned with a positive cycle of using natural resources and a negative cycle of emissions as potential contributors to climate change. The interaction with society results in positive feedbacks such as technological innovations and workforce development although negative feedbacks in terms of possible labor strikes (disruptions at SC resilience level) or global pandemics (disruptions at SC survivability level) also exist.

In summary, the VSC framework integrates the angles of sustainability and resilience, extending them by survivability, and offers a VSC model of interactions between SCs and their ecosystems at the levels of profitability, resilience, and survivability.

#### Viable supply chain model

With the development of the VSC model, we argue in favor of multiple structural SC designs for matching supply and demand according to the three feedback cycles of viable SC ecosystem framework and, most importantly, establishment and control of adaptive mechanisms for transitions between the structural designs. The rationale behind several structural designs for matching supply and demand stems from three feedback cycles shown in Sect. 2.2.1, i.e.:

- agility-oriented cycle,
- resilience-oriented cycle, and
- survival-oriented cycle.

Most importantly, SCs need established and manageable adaptive mechanisms for fast transitions between three structural designs (Fig. 3).

In Fig. 3, the VSC model is presented spanning four perspectives, i.e., structural view, dynamic state view, performance view, and control view. The structural view represents three SC structures which are activated through adaptation and recovery actions (note that the mathematical symbols used in Fig. 3 will be discussed later in Sect. 3). Here we follow the research stream on structural SC adaptations (Allesina et al. 2010; Ivanov et al. 2010; Pavlov et al. 2019a). The VSC structural view can be exemplified as follows. Consider a car manufacturer with a global SC. This SC can have one structural design at the times of economic stability and growth with full utilization of global sourcing, lean and agility advantages, offering a broad variety of products to satisfy individual customer needs (Dubey et al. 2018). The second structural design relates to the disruptions and maintaining the resilience in case of singular, local events such as natural disasters, strikes, fires, etc. This kind of design, i.e., the resilient SC builds around proactive and reactive capabilities such as risk mitigation inventory, capacity flexibilities, and backup suppliers (Hosseini et al. 2019). Finally, in cases of long-term, global disruptions such as COVID-19 pandemic, the third SC design is adapted which might be characterized by production changeovers (e.g., production of ventilators or masks instead of cars), reducing the product variety, radical changes in supplier base and logistics, and production localization (Simchi-Levi 2020).

We note that it might be a very challenging task to operate and control three SC designs simultaneously, both in terms of efficiency and complexity. In addition it is nearly impossible to predict all possible future scenarios and respective SC designs for matching supply and demand in these scenarios. As such, the main role in the VSC belongs to adaptation and recovery mechanisms, their design, establishment, training and implementation. It might be instructive for firms to “virtually” design and simulate the SC structures for resilience and survivability, and focus on the adaptation trainings to practice the SC changeability.

The state view of the VSM represents the adaptation and recovery processes in time in a two-dimensional state space. Both axes represent the transitions of SC structure (i.e., network configuration) in time. The states X_1_ depict the leagility level, X_2_–the resilience level, and X_3_–the survivability level. The horizontal transitions [e.g., between X_1_(S_11_) and X_1_(S_12_)] reflect the SC configuration changes within the same layer (e.g., the leagility). These structural transformations happen, for example, at the leagility level due to the re-designing a supplier base or a distribution network based on profit improvements or cost reduction activities. The vertical transitions between the states are triggered by adaptation against disruptions and recovery. We note that the structures and states can have a single dimension representing SC configurations in the form of a network design as composed of different organizations and also be considered from the multi-structural point of view combining organizational, informational, financial, product, and process-functional structures. We refer to the study by Ivanov et al. (2010) for more information about multi-state, multi-structural SC design.

The performance view in Fig. 3 suggests an illustration of the SC performance reaction to the stressors of different severity according to three levels coined in the structural view. The technical part of the VSC model, i.e., the dynamic state and control views will be discussed in detail in Sect. 3. All these four perspectives, i.e., structural view, dynamic state view, performance view, and control view provide as an integral whole a comprehensive description of a VSC model.

#### Multi-structural VSC view

Distinctively, the VSC elements can be presented from the multi-structural SC perspective (Fig. 4).

Literature analysis allows for identifying intersections of leagility, resilience, sustainability, and digitalization. Though these interrelations have not been brought into an integrity so far which is a substantial and distinctive contribution made by the multi-structural VSC view. The studies by Dubey et al. (2018), Gunasekaran et al. (2018), Ivanov and Dolgui (2019), and Zhong et al. (2017) elaborate on interrelations between lean, agile, and digital for increasingly data-driven market responsiveness. Efficient and resilient SCs (so-called LCN, low-certainty-need SCs) with the advantages of both lean and risk-resistant/recoverable SCs have been studied by Ivanov and Dolgui (2019). The studies by Amindoust (2018), Ivanov (2018a), Fahimnia et al. (2018), Fiksel (2003), and Ramezankhani et al. (2018) develop an integrated resilience-sustainability perspective. Papadopoulos et al. (2017) and Manupati et al. (2020) present the insights on the mutual relations between digital technologies and sustainability. The interface of digital and resilient SC has been studied by Cavalcantea et al. (2019); Choi et al. (2017); Choi and Lambert (2017); Dubey et al. (2019b); Ivanov (2017b); Ivanov et al. (2019a), and Ivanov and Dolgui (2020b). Altay et al. (2018); Dubey et al. (2019b), and Ivanov et al. (2018a) organized a debate around the intersections of flexibility, agility and uncertainty and developed the discussion towards the roles of agility and flexibility in achieving SC resilience. Fahimnia et al. (2014) and Dubey et al. (2015) pointed to empirically revealed intersections of the leagility and sustainability. Galaitsi et al. (2020) present empirical insights on the relations between system performance concepts such as adaptability, agility, reliability, resilience, resistance, robustness, safety, security, and sustainability.

The VSC model extends the existing state-of-the-art SC resilience knowledge and builds upon the resilient mechanisms that have been extensively discussed and classified in the studies by Bier et al. (2020), Ho et al. (2015), Hosseini et al. (2019a), Ivanov et al. (2017), Melnyk et al. (2014), Pettit et al. (2019), Snyder et al. (2016), and Tukamuhabwa et al. (2015), to name a few. In addition, literature on the SC ripple effect allows deducing the SC viability antecedents and drivers (Ivanov et al. 2014a, b; Garvey et al. 2015; Dolgui et al. 2018; Levner and Ptuskin 2018; Scheibe and Blackhurst 2018; Hosseini and Ivanov 2019; Ivanov et al. 2019a; Kinra et al. 2020; Li et al. 2019; Mishra et al. 2019; Sinha et al. 2020; Dixit et al. 2020; Dolgui et al. 2020b; Garvey and Carnovale 2020; Goldbeck et al. 2020; Li and Zobel 2020; Özçelik et al. 2020). Most of the SC resilience studies build their arguments around capacity and inventory reservations as well as back-up suppliers to cope with SC disruptions (Behzadi et al. 2018; Chen et al. 2011; Lücker et al. 2017, 2019; Hosseini et al. 2019b; Ivanov and Rozhkov 2017; Paul et al. 2019; Paul and Rahman 2018; Sawik 2016, 2019; Schmitt et al. 2017; Song et al. 2018; Spiegler et al. 2016, 2017; Yin and Wang 2018; Yoon et al. 2018). Some studies extended the operational discussion toward the product substitution and process/product modularity as mitigation and recovery policies (Lu et al. 2011; Gupta and Ivanov 2020). In addition, a few studies investigated structural and operational dynamics in the SC in an integrated manner (Ivanov and Sokolov 2019; Dolgui et al. 2020b). Finally, the developments in digital technologies have been utilized in SC resilience research (Baryannis et al. 2019; Dubey et al. 2019b; Ivanov et al. 2019b; Queiroz et al. 2019; Ivanov and Dolgui 2020a; Fragapane et al. 2020) pointing to the contributions of digital technology to SC viability in the areas of improving demand forecasting by data analytics, production flexibility by additive manufacturing, and SC visibility using digital twins.

### On the relations between supply chain resilience and viability

After the publication of our recent study on the viability of intertwined supply networks (Ivanov and Dolgui 2020b) and throughout the review process of this article, we have had several discussions with risk management and engineering experts on the relations of resilience and viability. We now lay out some relevant aspects which can be considered. We note that our discussion relates to SC resilience and viability, and not related to the resilience and viability in general, since the understandings differ across the disciplines [e.g., engineering, information, ecology, and medical sciences (Hosseini et al. 2016; Linkov and Kott 2019)].

To start, we suggest considering SC resilience definitions. For example, 13 different SC resilience definitions are presented in (Hosseini et al. 2019). When summarizing these definitions, we can observe that resilience has been discussed in terms of withstanding the disruptive *events* and recovery to a robust state of operations and normal *performance*. From this analysis, we can conclude that resilience is the main part of SC viability. There is no doubt that resilience will play a leading role guaranteeing viability of the SCs of the future. Having said that, with viability we understand the following combination of features, i.e.:

- Evolution and adaptation of the SC structures and processes in time,
- Multiple feedback cycles (i.e., leagile states—disrupted states—survival states), and
- Survivability over time as the major assessment criterion.

As such, viability takes a more generalized perspective seeking to encompass different conditions surrounding SCs over long time horizons and develop adaptive network structures and strategies to guide the SCs facing both positive and negative changes in the environments. One key issue in resilience is that it is mostly related to system reactions to negative events (e.g., absorb-recover-adapt). Viability considers both positive events (i.e., market growth, profitability) and negative events (disruptions) because the SCs experience both positive and negative events through their lifecycles. Resilience is a central and leading focus of viability responsible for protection, adaptation and recovery. If a SC is able to withstand disruptions, ripple effects and recover, this SC is resilient. If a resilient SC is able to maintain itself and survive at a long-term perspective in changing environments over the whole lifecycle through adaptation of supply–demand structures and performances, then we talk about a *viable SC*. We note that an assessment of SC viability can be related to the ability of providing certain products or services for markets and societies in the required scope and at a long-term scale.

#### *Example 1*

To illustrate, consider an example of an automotive SC. From the positions of resilience, a car manufacturer can establish an SC with some backup facilities, inventory buffers, flexible capacities, and a visibility control system to enable the robustness and recovery against, e.g., severe natural disasters which may temporarily, adversely affect in- and outbound material flows. The resilience would be assessed by a performance of the car manufacturer, e.g., annual revenues or service level. From the positions of viability, the SC of the car manufacturer should ensure leagility and profitability, be resilient and deliver the mobility service to society at a long-term perspective.

#### *Example 2*

Another way to exemplify the relations between resilience and viability is to consider a living organism. In our understanding, SC resilience is close to the role of immune systems, and viability is the ability to survive over the whole life through absorbing negative impacts with the help of a strong immune system and achieving performance by using positive chances. A strong immune system and acting in line with nature and society help human beings achieving high performances. Similarly, the SC performance depends on the resilience and sustainability. A weak immune system may result in performance degradation. Low SC resilience, if a disruption is experienced, also results in profitability reductions, mismatches of demand and supply, and destabilization of normal operations. Continuing the analogy, immune systems of each human being are in a continuous interaction with the environment. This property is reflected by viability in terms of utilizing positive feedbacks with the environment (e.g., making profits in growing markets), sustaining against negative impacts (e.g., facility disruptions) and surviving in a case of substantial changes in the environments, e.g., global pandemics. Put simply: strong immune systems help the living organisms to live and survive; strong resilience helps SCs to perform over the whole lifecycle and under different positive and negative conditions, i.e. to be viable.

#### *Remark on short- and long-term understanding of SC viability*

Viability is a convenient concept to address both “acute” issues of surviving under very severe stressors such as global pandemics and “chronic” concerns about guiding an SC through its whole lifecycle in the long-term perspective.

## Formal model of SC viability

In this section, we illustrate the VSC model using dynamic systems theory and SC structural dynamics control approach (Ivanov and Sokolov 2010; Ivanov et al. 2010; Ivanov 2018b and Ivanov and Sokolov 2019).

At the levels of structure and state dynamics (cf. Fig. 3), let $S = \left\{ {\;S_{\chi } ,\chi \in N} \right\}$ be the set of SC structures formed through adaptation and recovery. In the example in Fig. 3, we consider three structures S_1_ $(\chi = 1)$, S_2_ $(\chi = 2)$, and S_3_ $(\chi = 3)$. In a generalized case, *N*-structures can exist. The structures can be described in an interconnected way using a *dynamic alternative multigraph* (Eq. 1):

1 $$ S_{\chi }^{t} = \left\langle {B_{\chi }^{t} ,F_{\chi }^{t} ,Z_{\chi }^{t} } \right\rangle , $$

where each point of time *t* belongs to an interval $t \in \left( {T_{0} ,T_{f} } \right]$; $B_{\chi }^{t} = \left\{ {x_{{\left\langle {\chi ,l} \right\rangle }}^{t} ,\;l \in L_{\chi } } \right\}$ is the set of elements of the structure $S_{\chi }^{t}$ (i.e., suppliers and manufacturers in an SC design) at point of time *t*; $F_{\chi }^{t} = \{ f_{{ < \chi ,l,l^{\prime} > }}^{t} ,l,\quad l^{\prime} \in L_{\chi } \}$ is the set of arcs (i.e., logistics in an SC design) at point of time *t*; $Z_{\chi }^{t} = \{ z_{{ < \chi ,l,l^{\prime} > }}^{t} ,l,\quad l^{\prime} \in L_{\chi } \}$ is the set of parameters that characterize the elements in an SC design numerically (e.g., capacities and inventories) at point of time *t*.

The graphs of different structures are interdependent thus, for each operation, the following maps should be constructed (Eq. 2):

2 $$ MM_{{ < \chi ,\chi^{\prime} > }}^{t} :F_{\chi }^{t} \to F_{{\chi^{\prime}}}^{t} . $$

Composition of the maps can be also used at point of time *t* as shown in Eq. (3):

3 $$ MM_{{\left\langle {\chi ,\chi^{\prime}} \right\rangle }}^{t} = MM_{{\left\langle {\chi ,\chi_{1} } \right\rangle }}^{t} \circ MM_{{\left\langle {\chi_{1} ,\chi_{2} } \right\rangle }}^{t} \circ \ldots \circ MM_{{\left\langle {\chi^{\prime\prime},\chi^{\prime}} \right\rangle }}^{t} . $$

The adaptations of SC structures can be described with the help of multi-structural states as the following inclusion (Eq. 4):

4 $$ {\text{S}}_{\chi } \subseteq {\text{X}}_{1}^{\text{t}} \times {\text{X}}_{2}^{\text{t}} \times {\text{X}}_{3}^{\text{t}} \times \ldots \times {\text{X}}_{{\left( {\text{n,m,k}} \right)}}^{\text{t}} ,\quad \chi = 1, \ldots ,{\text{N}}, $$

where *n, m, k* are the total numbers of SC structural states at the levels of leagility, resilience and survivability, respectively.

Now we obtain the set of the SC multi-structural states in dynamics (5):

5 $$ {\text{S}} = \left\{ {{\text{S}}_{\chi } } \right\} = \left\{ {{\text{S}}_{{1\left( {\text{n,m,k}} \right)}} , \ldots ,{\text{S}}_{{{\text{N}}\left( {\text{n,m,k}} \right)}} } \right\}. $$

With the help of mapping, we now describe the allowable transitions between the states both in adaptation and recovery directions (Eq. 6):

6 $$ \Pi_{ < \chi ,\chi ' > }^{t} :S_{\chi } \to S_{\chi '} . $$

Assuming that each multi-structural state at time $t \in (T_{0} ,T_{f} ]$ is defined by a composition (6), we now formulate the problem of VSC control over time as shown in Eqs. (7) and (8):

7 $$ J_{\zeta } \left( {B_{\chi }^{t} ,F_{\chi }^{t} ,Z_{\chi }^{t} ,MM_{{ < \chi ,\chi^{\prime} > }}^{t} ,\Pi_{{ < \chi ,\chi^{\prime} > }}^{t} ,\;t \in (T_{0} ,T_{f} ]} \right) \to \mathop {extr}\limits_{{ < U^{t} ,S_{\chi }^{{*T_{f} }} > \in \Delta^{(d)} \cup \Delta^{(s)} }} , $$

8 $$ \begin{aligned} \Delta^{(d)} \cup \Delta^{(s)} & = \left\{ {\left. {\left\langle {U^{t} ,S_{\delta }^{{T_{f} }} } \right\rangle } \right|R_{{\tilde{r}}} } \right.\left( {X_{\chi }^{t} ,F_{\chi }^{t} ,Z_{\chi }^{t} ,MM_{{\left\langle {\chi ,\chi^{\prime}} \right\rangle }}^{t} ,\Pi_{{\left\langle {\tilde{\delta },\tilde{\tilde{\delta }}} \right\rangle }}^{t} } \right) \le \tilde{\tilde{R}}_{{\tilde{r}}} ; \hfill \\ & \quad \left. {U^{t} = \Pi_{{\left\langle {\delta_{1} ,\delta_{2} } \right\rangle }}^{{t_{1} }} \circ \Pi_{{\left\langle {\delta_{2} ,\delta_{3} } \right\rangle }}^{{t_{2} }} \circ \ldots \circ \Pi_{{\left\langle {\tilde{\delta },\delta } \right\rangle }}^{{t_{2} }} } \right\}, \hfill \\ \end{aligned} $$

where $U^{\,t}$ are control actions for SC adaptation and recovery, $J_{\zeta }$ are SC performance indicators (e.g., costs and service level), $\zeta \in \left\{ {1, \ldots ,\Im } \right\}$ is the set of the performance indicators, $\Delta^{(d)} \cup \Delta^{(s)}$ is the set of dynamic (*d*) and static (*s*) alternatives of SC network designs, $\tilde{r} \in \left\{ {1, \ldots ,\tilde{R}} \right\}$ is the set of material and information processes, $R_{{\tilde{r}}}$ is the set of constraints on material and information processes; $\tilde{\tilde{R}}_{{\tilde{r}}}$ are constants, which are known and $t = (T_{0} ,\,T_{f} ]$ is time horizon. Other symbols have been explained above and used in line with (Ivanov and Sokolov 2010).

At the control level (cf. Fig. 3), the SC input–output dynamics is described by state vector ${\dot{\mathbf{x}}}_{p1} (t)$, control vector u(t), perturbation vector $\xi (t)$, recovery control vector u_c_(t), and output performance vector ${\mathbf{y}}(t)$. For the general case this dynamic can be described as shown in Eqs. (9) and (10):

9 $$ {\dot{\mathbf{x}}}_{p1} (t) = f({\mathbf{x}}(t),\,{\mathbf{u}}(t),\,{\varvec{\upxi}}(t),\,\alpha ,{\varvec{\upbeta}},t) $$

10 $$ {\mathbf{y}}(t) = f\left( {{\mathbf{x}}(t),{\mathbf{u}}(t),{\varvec{\upxi}}(t),\alpha ,{\varvec{\upbeta}},t} \right) $$

The state control vector ${\dot{\mathbf{x}}}_{p1} (t)$ represents the SC according to the state dynamics shown in the upper, right-hand part of Fig. 4. The ${\mathbf{y}}(t)$ is measured by a monitoring system *F* with regards to compliance with the input state variables $x_{pl} (t)$ Based on information feedbacks in *F* and disruption data $\xi$, deviation of output performance from plan ε = $\left\| {{\mathbf{y^{\prime}}}(t) - \left. {{\mathbf{y}}_{pl} (t)} \right\|} \right.$ is computed, and an adapted input $u_{c} (t)$ is generated for recovery control actions ***u***_c_(*t*) .

SC performance evaluation can be described by Eq. (11):

11 $$ {\mathbf{J}}_{\Theta } \left( {{\mathbf{x}}(t),{\mathbf{u}}(t),{\varvec{\upxi}}(t),{\varvec{\upbeta}},t} \right) = \left\| {\mathbf{J}} \right\|^{\text{T}} $$

In the right-hand part of Fig. 4, we can distinguish four control scenarios (corresponding to arrows outgoing from the F-block and the SC states):

- the planned operation policy ${\mathbf{x}} = {\mathbf{f}}(t,{\mathbf{x}},\,{\mathbf{u}})$ can be executed despite a disruption (i.e., the stability case)
- the planned operation policy ${\mathbf{x}} = {\mathbf{f}}(t,{\mathbf{x}},\,{\mathbf{u}})$ can be executed despite a disruption using some SC redundancy (i.e., the robustness case)
- disruptions affect the SC operations, the components of **J**_**Θ**_^T^ deviate from the plan, but the ${\mathbf{x}} = {\mathbf{f}}(t,{\mathbf{x}},\,{\mathbf{u}})$ can be recovered (i.e., the case of resilience)
- disruptions affect the SC operations, the components of **J**_**Θ**_^T^ deviate from a plan, no recovery program **v(t)** can be found to offer an updated ${\mathbf{x}}_{\sigma } = {\mathbf{f}}(t,{\mathbf{x}},\,v)$ in order to achieve the planned **J**_**Θ**_^T^. In this case, the SC viability should be analysed.

For further technical details of the SC viability control, we refer to the studies on the SC multi-structural control (Ivanov et al. 2010), attainable sets for SC viability assessment (Ivanov et al. 2018b), and recovery control (Ivanov and Sokolov 2019).

## Future research directions

Future research in SC viability can be organized around multiple perspectives. In this section, we summarize several future research directions.

### Intertwined supply networks (ISN)

SCs evolve towards ISNs (Ivanov and Dolgui 2020b) that are characterized by structural dynamics. Different from linearly directed SCs with static structures, the firms in ISNs may exhibit multiple behaviors in buyer–supplier relations (i.e., behavioral dynamics) in interconnected or even competing SCs simultaneously. These new dynamic, co-evolving structures require rethinking of some traditional analysis concepts and could be very interesting in regard to SC viability. For example, the COVID-19 pandemic clearly showed complex, and at times unforeseen interconnections between industrial, healthcare, pharmaceutical, and food SCs. As such, novel, cross-sectoral and adaptable SC designs can be examined in future. This research avenue will be supplemented by investigations in flexible production/service technologies and human–robot collaborations for timely reactions to changing environments and switching between three feedback cycles of the VSC model (e.g., a switch from car manufacturing at “normal” times to ventilator production during a pandemic). Obviously, these adaptable structures and technologies are expected to be supported by innovative product and facility designs engaging all actors in the SC ecosystems from public and private sectors.

### Digital twins and data-driven technologies

Another promising research area for SC viability is the utilization of digital, data-driven technologies to uncover their potential in decision-making support in cases of long-term, severe disruptions such as pandemics. In particular, digital SC twins (Ivanov and Dolgui 2020a)—the computerized SC models that represent the network state for any given moment in real time—can be further investigated in this direction in order to examine the role and value of information collection, data analytics, mapping and coordination in SC viability. Other interesting concepts for framing SC viability, its antecedents, its drivers, and its economic and social performance implications are LCN (low-certainty-need SCs) as a trade-off between efficiency and resilience, ripple effect and SC viability, ecological modelling, and RSC (reconfigurable SCs).

### Multi-methodological analysis

From the methodological point of view, SC viability analysis offers a room for almost all quantitative and empirical methodologies. For example, optimization techniques can be used for multi-level SC design (cf. Fig. 3). Simulation methods can be applied to recovery analysis (Ivanov 2020a). Ecological modelling can shed light on underlying behaviors of collective survivals (Demirel et al. 2019, Ivanov and Dolgui 2020b). Bayesian networks can help in analyzing causal relationships and disruption propagations (i.e., the ripple effect) in the networks (Garvey et al. 2015, Hosseini et al. 2019). Interactions in the intertwined SCs and ecosystems would open new problem settings for game-theoretic studies. We also see new applications for control theory in the area of SC viability due to feedback and dynamics considerations (Ivanov and Sokolov 2013). A specific role can be played by hybrid data-driven approaches blended with optimization.

## Conclusions

Viability is an ability of an SC to maintain itself and survive in a changing environment, with the redesign of the structures and replanning economic performance with long-term impacts. In this paper, we theorized a new notion—the Viable Supply Chain (VSC). Our approach integrates the angles of sustainability and resilience, extending them by survivability, and offers a VSC framework within SC ecosystems. The principal ideas of the VSC model are multiple structural SC designs for supply–demand matching and, most importantly, establishment and control of adaptive mechanisms for transitions between the structural designs. We argue in favor of adaptable networks that exhibit the features of leagility, resilience against disruptions, and pandemic-resistance.

Further, we demonstrate how the VSC components can be categorized across organizational, informational, process-functional, technological, and financial structures. Through the lens and guidance of dynamic systems theory, we illustrate the VSC model at the technical level. Definitely, other methodologies can be applied to investigate different aspects of VSCs such as mathematical optimization, discrete-event simulation, agent-based modeling, system dynamics, game theory, ecological modelling, Bayesian networks, to name a few. Moreover, VSC principles can be further extended in the framework of intertwined supply networks (ISN) and using data-driven, digital technologies.

The VSC model can be of value for decision-makers to design SC structures, processes, information and financial systems that can be profitable during the positive times, able to withstand disruptions and recover, and survivable during long-term, global disruptions with societal and economic shocks. In future, managerial insights of different VSC applications can be studied and articulated across different industries and services, spanning entire SC ecosystems.

To summarize, the SC and operations management community has created impressive methodical fundamentals, techniques and tools for leagility, resilience, sustainability and digitalization of SCs for the last three decades. The COVID-19 pandemic has revealed a series of novel challenges for SC and operations management which beget an understudied research area—SC viability—which builds upon and extends to an integral whole the angles of leagility, resilience, sustainability and digitalization. Substantial contributions can be expected in this regard in almost all the existing areas of SC and operations management, and new research streams can emerge.
