# Correction to: Viable supply chain model: integrating agility, resilience and sustainability perspectives—lessons from and thinking beyond the COVID-19 pandemic

**DOI:** 10.1007/s10479-021-04181-2

**Published:** 2021-07-26

**Authors:** Dmitry Ivanov

**Affiliations:** Department of Business Administration, Berlin School of Economics and Law, 10825 Berlin, Germany

## Correction to: Annals of Operations Research 10.1007/s10479-020-03640-6

The article Viable supply chain model: integrating agility, resilience and sustainability perspectives—lessons from and thinking beyond the COVID-19 pandemic, written by Dmitry Ivanov, was originally published electronically on the publisher’s internet portal on 22 May, 2020 without open access.

With the author(s)’ decision to opt for Open Choice the copyright of the article changed on 22 June, 2021 to © The Author(s) 2021 and the article is Open Access This article is licensed under a Creative Commons Attribution 4.0 International License, which permits use, sharing, adaptation, distribution and reproduction in any medium or format, as long as you give appropriate credit to the original author(s) and the source, provide a link to the Creative Commons licence, and indicate if changes were made.

The images or other third party material in this article are included in the article’s Creative Commons licence, unless indicated otherwise in a credit line to the material. If material is not included in the article’s Creative Commons licence and your intended use is not permitted by statutory regulation or exceeds the permitted use, you will need to obtain permission directly from the copyright holder. To view a copy of this licence, visit http://creativecommons.org/licenses/by/4.0/

